# Avasopasem manganese acts as both a radioprotector and a radiomitigator of radiation-induced acute or late effects

**DOI:** 10.3389/fonc.2026.1761003

**Published:** 2026-02-20

**Authors:** Brock J. Sishc, Deepti Ramnarain, Zengfu Shang, Elizabeth M. Alves, David A. Bloom, Kelly Hughes, Debabrata Saha, Dennis P. Riley, Jeffrey L. Keene, Robert A. Beardsley, Michael D. Story

**Affiliations:** 1 Division of Molecular Radiation Biology, Department of Radiation Oncology, University of Texas Southwestern Medical Center, Dallas, TX, United States; 2 Department of Veterinary Medicine, Colorado State University, Ft Collins, CO, United States; 3 Division of Molecular Radiation Biology, Department of Radiation Oncology, Galera Therapeutics, Malvern, PA, United States; 4 Simmons Comprehensive Cancer Center, University of Texas Southwestern Medical Center, Dallas, TX, United States

**Keywords:** chromosomal integrity, lung fibrosis, mutation, oral mucositis, radiation, radio mitigation, radioprotection

## Abstract

**Introduction:**

The pentaazamacrocyclic superoxide dismutase mimetic, Avasopasem Manganese (AVA), has been shown in clinical trials to reduce the severity and duration of acute oral mucositis (OM) and acute esophagitis in patients treated for head and neck and lung cancers, respectively, by radiotherapy using conventional fractionation protocols. Here, the radioprotective effects of AVA were tested in normal tissues after high dose per fraction radiation exposures to determine whether: radioprotective effects of AVA were still present after doses like those used with stereotactic ablative radiotherapy (SAbR); AVA protected against late normal tissue responses; and, whether AVA could act as a radiomitigator of adverse normal tissue events.

**Methods:**

With AVA, residual DNA lesions and micronuclei were reduced in HBEC3 KT but increased in H1299 cells 24h post-irradiation. Furthermore, radiation-induced mutations and chromosome aberrations were reduced in WTK-1 lymphoblast cells. The radioprotective effects of AVA at high dose per fraction were then tested against both acute and late normal tissue effects.

**Results:**

When provided prior to radiation, AVA reduced the extent of epithelial cell layer degradation of mouse tongue irradiated with a single dose of 17 Gy and reduced radiation recall when a second dose of radiation of 12 or 17 Gy was given two weeks following the initial 17 Gy dose. In addition, when provided after radiation, there was a modest but significant reduction in adverse epithelial layer response. Radiation-induced lung fibrosis, determined at 24 weeks post-irradiation, was also reduced when AVA was delivered 1 hour prior to irradiation after a single dose of 54 Gy. When AVA was provided 24h after 54 Gy and given daily (Monday-Friday) for increasing numbers of weeks, fibrosis was progressively reduced as the length of AVA treatment increased. However, AVA’s effect on fibrosis decreased as the time between irradiation and post-irradiation AVA application increased.

**Conclusions:**

These studies confirm the efficacy of AVA as a radioprotector and mitigator of both radiation-induced acute and late effects after high dose per fraction exposures while not protecting tumor cells to radiation exposure.

## Introduction

From its formative years in becoming a major therapeutic modality for cancer therapy, radiotherapy (RT) has sought to maximize the probability of tumor control by delivering as much dose to the tumor as possible, while limiting the dose to non-tumor tissues, thus reducing the likelihood of what can be life-threatening adverse normal tissue events (1). Indeed, tumor doses are driven by tolerable doses to the surrounding normal tissue. Maximizing the therapeutic ratio between tumor and normal tissue still drives modern RT, especially as RT moves into an era of near-marginless targeting of tumors while utilizing ablative, high-dose-per-fraction doses of radiation, where there is the risk for catastrophic adverse normal tissue events. Acute adverse events, those that occur during RT or shortly thereafter, can be life-threatening, can halt or interrupt a therapeutic regimen, and can lead to a diminished quality of life for those whose cancer therapy was successful. Adverse events that occur months later (late effects) can also be deleterious and life-shortening and can negatively impact long-term quality of life. Further, concerns for quality of life are increasing given the overall increase in lifespan following the successful completion of cancer therapy (2).

Acute effects are generally believed to result from the direct killing of cell populations with rapid turnover rates, such as those of the oral mucosal epithelial cells or the crypt cells of the intestinal epithelium, thereby leading to inflammatory responses and ulceration (3). Of particular concern for the treatment of squamous cell carcinoma of the head and neck (HNSCC) is radiation-induced oral mucositis (OM), which generally begins mid-treatment and includes painful, severe ulceration in the oral cavity that can impair nutrition and hydration, require tube feeding and hospitalization, and lead to pause or discontinuation of RT, compromising tumor response. Indeed, discontinuation of therapy is a negative prognostic indicator of therapy. Furthermore, OM can last for weeks to months after treatment is completed (4). Another example of an acute effect is radiation pneumonitis, which is excessive inflammation of the lung due to RT; however, this consequence is not generally treatment-limiting, as it is well controlled with steroid administration (5).

Late effects, in contrast, are generally thought to be driven by the inherent biological response to radiation damage, such as chronic inflammation and oxidative stress, leading to consequences appearing months to years later. An example of such a late effect would be radiation-induced lung fibrosis (RILF), which involves thickening of the lung tissue with decreased breathing capacity and diminished cardiac function. RILF is a primary factor limiting the amount of radiation dose that can be delivered to a given volume of the lung during the treatment of lung cancer (6).

A strategy to limit the risk of RT-related side effects is utilizing radiomodifiers, pharmacological compounds that alter the biological response of tissues to ionizing radiation (IR) exposure. These fall into two categories: radioprotectors and radiomitigators. Radioprotectors must be present in tissues at appropriate concentrations at the time of radiation and function by dampening the damage from the initial burst of radiation. One proven approach is targeting the burst of reactive oxygen species (ROS) produced by IR via the radiolysis of water in a tissue. To date, the only Federal Drug Administration (FDA)-approved radioprotector is amifostine, which functions by scavenging ROS; however, safety and pharmacokinetic concerns restrict its use in most settings (7). Radiomitigators, however, target the radiation response processes that promulgate damage long after the IR exposure occurred and therefore do not need to be present at the time of irradiation. There is currently no FDA-approved radiomitigator in the radiotherapy setting; however, mitigators exist to reduce the lethality of acute radiation syndrome (ARS), specifically hematopoietic ARS, by stimulating hematopoietic stem cell mobilization to limit the increased risk of infection due to the loss of lymphatic cells (8). A third type of radiomodifier, radiosensitizers, is also in development; however, these are primarily designed to sensitize tumors to IR, in effect lowering the required tumor dose without the loss of efficacy and thus reducing dose to adjacent at-risk normal tissue. Importantly, for a radioprotector or a radiomitigator to be considered effective, it should not protect the tumor (9). Furthermore, an ideal radiomodifier would protect or mitigate normal tissue damage while also increasing tumor control, as has been described for avasopasem manganese (AVA) (10).

A new family of agents, pentaazamacrocyclic manganese superoxide dismutase mimetics (Galera Therapeutics, Malvern, PA, USA) represented by AVA, have demonstrated promise as radioprotectors by the dismutation of the damaging ROS superoxide into hydrogen peroxide, which is detoxified by endogenous catalase and peroxidase enzymes into water (11, 12). AVA catalyzes this conversion at a similar rate to endogenous superoxide dismutase (SOD) enzymes and is a true catalyst in the sense that it is not consumed in the dismutation reaction.

AVA not only has an excellent safety profile, but it has also demonstrated success at reducing both the incidence and duration of World Health Organization (WHO) radiation-induced severe (grade 3 and 4) oral mucositis, or SOM, in trials of patients undergoing conventionally fractionated (2 Gy/fraction) chemoradiation therapy (CRT) for the treatment of HNSCC (NCT04476797) (13, 14). More recently, a phase III, randomized, double-blind, placebo-controlled trial enrolling 455 patients across 97 institutions undergoing CRT for the treatment of HNSCC again demonstrated a reduction in the duration and a delay in the onset of radiation-induced SOM (15, 16). Additionally, in a phase 2, open-label trial examining radiation esophagitis in patients receiving chemoradiation therapy for non-metastatic lung cancer, AVA resulted in only 3% of patients developing WHO grade 3 or higher esophagitis (NCT04225026) (17).

In addition to the demonstrated clinical efficacy of AVA for radiation-induced SOM, some preclinical and clinical evidence suggest that AVA is not only an effective radioprotector in the context of radiation-induced OM, but it also does not protect tumors from RT. In fact, when utilized in conjunction with stereotactic ablative radiotherapy (SAbR) [radiation doses of 8 Gy or higher given in five or fewer fractions, also called stereotactic body radiotherapy (SBRT)], AVA enhanced tumor response to irradiation in preclinical models of lung, pancreatic, and HNSCC tumors (10). Moreover, in a phase 1b/2 clinical trial of AVA combined with SBRT for borderline resectable or locally advanced pancreatic cancer, there were increases in tumor response, progression-free survival, and overall survival in the AVA arm compared to the control arm (18).

Several questions remain regarding the potential of AVA. First, if AVA is effective as a radioprotector capable of reducing both the incidence and severity of radiation-induced OM, an acute effect, will it also be effective at reducing RT-induced late effects? Second, considering that molecular processes driving radiation-induced damage also involve ROS generated well beyond that produced in the initial radiochemical events, as a ROS scavenger, does AVA have potential radiomitigative properties? Third, while AVA has potent anti-tumor properties when combined with SAbR and does not protect tumors under conventionally fractionated regimens, are oncogenically progressed cells protected from radiation, which could, with time, allow those cells to become tumorigenic? Finally, as SAbR is commonly used as a salvage therapy for recurrent disease, the phenomenon of “radiation recall” is a concern. This is defined as a “remembrance” of radiation damage yielding acute toxicities at lower radiation doses or with alternative cytotoxic or immunologic agents (including immune checkpoint blockades) (19). Using AVA during primary treatment may therefore be effective at limiting radiation recall or reducing mucositis in the re-irradiation setting.

These open questions are addressed in this study. First, the differential response of H1299 non-small cell lung cancer (NSCLC) cells and normal HBEC3 KT bronchial epithelial cells to the combination of radiation and AVA was evaluated. AVA H1299 cells demonstrated an increase in the number of persistent DNA damage foci and micronuclei following the combination of IR and AVA, while HBEC3 KT demonstrated a reduction in both these endpoints. Further, to test the ability of AVA to reduce mutational burden following irradiation, non-tumorigenic WTK1 cells were assayed to determine the percentage of TK6^−/−^ mutations induced by irradiation and the formation of chromosome aberrations, with AVA reducing both endpoints. Second, the efficacy of AVA as both a radioprotector and a radiomitigator in a preclinical model of late normal tissue effects, i.e., RILF, was tested. Some of these experiments were conducted in the context of a single high dose to test the limits of AVA to protect from RILF in a tissue volume that was closer to human treatment volumes as a percentage of total lung volume, demonstrating AVA’s normal lung radioprotection and radiomitigation from late, potentially chronic, toxicity after high-dose-per-fraction radiation. Third, results from preclinical studies of radiation-induced OM are described, also demonstrating AVA radioprotection and radiomitigation from early toxicity, as well as reduction in radiation recall after high-dose-per-fraction radiation. Given the results of the aforementioned clinical trials and the radio-enhancing properties of AVA when combined with SAbR, these results demonstrate that AVA is not only an effective radioprotector against radiation-induced OM and lung fibrosis after high-dose-per-fraction radiation but that it can also mitigate these toxicities when provided after radiation exposure. Furthermore, AVA protected cells from DNA damage, reduced mutational burden, and reduced IR-induced genomic instability, suggesting that it would not promote the oncogenic progression of potentially carcinogenic cells.

## Materials and methods

### Cellular and animal models

H1299 non-small cell lung cancer cells (CRL-5803, American Type Cell Culture (ATCC) repository) were cultured in RPMI 1640 basal cell culture medium (Corning, NY, USA) supplemented with 10% fetal bovine serum (FBS; Atlas Biologicals, Fort Collins, CO, USA). Oncogenically non-transformed, immortalized human bronchial epithelial (HBEC) HBEC-3KT cells were a kind gift from Dr. John Minna (University of Texas Southwestern Medical Center, Dallas, TX, USA) and cultured as described previously in Keratinocyte Serum Free Medium (Thermo Fisher, Waltham, Massachusetts, USA) (20–22). WTK1 lymphoblastoid cells were maintained in suspension culture using RPMI 1640 basal medium supplemented with 10% FBS (23). Eight-week-old female C57BL/6 and C3H mice were purchased from Charles River Laboratories (Charleston, SC, USA), and husbandry was conducted according to protocols approved by the Institutional Animal Care and Use Committee (IACUC) at the University of Texas Southwestern Medical Center (UTSW).

### Irradiation

*In vitro* cellular irradiations were conducted utilizing a Mark 1 ^137^Cs sealed source irradiator (J.L. Shepherd and Associates, San Fernando, CA, USA) at a dose rate of 3–4 Gy/min. Animal irradiations were conducted utilizing an X-rad 320 irradiator (Precision X-Ray, Madison, CT, USA) running at 250 kVp at 15 mA (24). Irradiations to mimic clinical SAbR protocols were conducted utilizing a Cx225 (Precision X-Ray, Madison, CT, USA) irradiator capable of image-guided treatment planning using an on-board CT. For a detailed breakdown of each irradiator’s usage, dosimetry, and field geometry, please refer to **Supplementary Table 2**.

### Isoflurane anesthetization and euthanasia

During irradiation, animals were immobilized using isoflurane inhalation at a 1%–2.5% concentration in oxygen. Animals were euthanized using CO_2_ inhalation at a flow rate sufficient to displace the volume of the container at a rate of 30%–70% per minute. After 5 minutes of asphyxiation, cervical dislocation was utilized as a confirmation of euthanasia.

### Immunostaining and micronucleus formation

HBEC3 KT cells were irradiated with 10 Gy of ^137^Cs γ-rays. At 24 hours post-irradiation, cells were fixed and stained for the presence of γH2AX and 53BP1 foci, representing DNA double-strand breaks (DSBs). Samples were stained and analyzed in biological triplicate (irradiated separately) and pooled for analysis. Foci in over 100 cells were counted for each replicate. In addition, more than 200 were evaluated for the presence of micronuclei, and the number of micronuclei per cell was reported for each treatment cohort (25).

### Genetic and genomic protection

To determine whether AVA altered the mutational burden of surviving cells, the mutation status of the thymidine kinase (TK) gene was tested using the WTK1 lymphoblastoid cell line that harbors a heterozygous mutation in the TK locus that makes it an effective model to test for deleterious mutation production by radiation (23, 26). The assay was performed in biological triplicate (cell cultures plated 48 hours prior to irradiation), and samples were irradiated separately and plated separately for mutational analysis. Error bars represent the SEM of three biological replicates. Additional information is included in the **Supplementary Methods**.

### Chromosomal aberration analysis

To determine whether AVA altered the induction of chromosome and chromatid aberrations 24 hours post-IR, AVA was added to the cultures of WTK1 cells 1 hour prior to a single acute dose of 4 Gy. Cells were allowed to incubate for 24 hours post-exposure, treated with Colcemid solution for 2.5 hours to enrich the percentage of cells in metaphase, and fixed to examine chromosome aberrations. The preparation of metaphase chromosome spreads and cytogenetic analysis were performed as previously reported (27, 28). Three independent cultures were irradiated per treatment group, and 50 metaphases were examined per biological replicate. Error bars represent the SEM of three independent biological replicates. Additional information is included in the **Supplementary Methods**.

### Protection from radiation-induced lung fibrosis

Ten-week-old female C57BL/6J mice were focally irradiated to the left lung using a 3-mm collimated X-ray beam to total doses of 54, 60, and 70 Gy in a single fraction. Targeted irradiation was performed using fluoroscopy. AVA was delivered once as a single 24 mg/kg i.p. injection delivered 30–60 minutes prior to irradiation. Lungs were perfused, and tissues were collected at time points of 4, 8, 12, and 24 weeks post-irradiation. Doses of 60 and 70 Gy were selected to determine if a dose threshold exists that limits the efficacy of AVA as a radioprotector, and tissues were collected at a time point of 12 weeks post-irradiation for histological analysis for these doses. Error bars represent the SEM of the mean, and the statistical unit of analysis was the individual animal.

### Modified Ashcroft scale

Due to the limited amount of lung volume irradiated, a modified Ashcroft scale was developed and used by a board-certified veterinary pathologist in a double-blind manner. A fibrosis score was generated according to the following scale and assessed using both Masson’s trichrome (MTC) and H&E-stained consecutive sections. For MTC-stained sections, 0 = no significant findings, 1 = <10% of the lung area affected, 2 = 10%–20% of the lung area affected with a single focal area, and 3 = 10%–20% of the lung affected with multiple focal lesions. For H&E-stained sections, 0 = no fibrosis, 1 = mild fibrosis, 2 = moderate fibrosis, and 3 = severe fibrosis.

### Semi-quantitative image analysis of fibrotic area

Semi-quantitative image analysis was utilized to provide an alternative independent method for establishing the extent and severity of fibrosis. The standard ImageJ package was utilized without the use of additional scripts, plugins, or macros to analyze ×40 Masson’s trichrome-stained images of murine lung as described previously (29). Image acquisition and image analysis were conducted by a board-certified veterinary pathologist, and images were blinded for independent analysis by a second individual. Briefly, full color images were gated to minimize all but blue (collagen)-stained regions, and a mask was applied to create a binary image. Gating threshold values were maintained throughout image analysis. Seven 100 × 100 µm squares were randomly placed throughout the field of three separate fibrotic frames from each animal, as well as three frames from the unirradiated portion of the lung of the same animal. Those numbers were averaged to measure an individual animal’s fibrotic area value and normalized against the density of the unirradiated lung. Individual animal fibrotic area measurements were averaged within each treatment group to generate the overall fibrosis score.

### Mitigation of radiation-induced lung fibrosis

Animals were focally irradiated using a single fraction of 54 Gy delivered with a 10-mm sphere of radiation dose using the X-Rad 320 irradiator (Precision X-Ray). AVA was delivered as a 24 mg/kg i.p. injection once prior to irradiation to replicate the protection studies. Additional treatment arms with AVA included delivery starting 24 hours post-irradiation and continuing as daily injections (Monday through Friday) for 1, 4, 8, 12, or 20 weeks post-irradiation or starting at 1, 4, 8, or 12 weeks post-irradiation and continuing until euthanasia at week 20. Error bars represent the SEM of the mean, and the statistical unit of analysis was the individual animal.

### Radiation-induced OM of the tongue

Based on previous studies, the snouts of 10-week-old female C3H mice were focally irradiated using a 10-mm collimated beam with a single acute dose of 17 Gy to cause radiation-induced OM in 100% of animals at 11–13 days post-irradiation (30). AVA was delivered i.p. as a 24 mg/kg injection i) once 30–60 minutes prior to irradiation, ii) daily starting 24 hours post-irradiation until euthanasia, or iii) once prior to irradiation and then daily until euthanasia. On day 11 post-irradiation, animals were euthanized; tongues were excised and stained with toluidine blue to determine where epithelial ulceration had occurred and then fixed in 10% neutral buffered formalin for 48 hours. Following fixation, tongues were embedded in paraffin and stained with H&E. Tongue sections were imaged using a Keyence BZ-X710 (Keyence, Minneapolis, MN, USA), and epithelial layer thickness was measured as described previously (31, 32). Error bars represent the SEM of the mean, and the statistical unit of analysis was the individual animal.

### Radiation recall

Animals were focally irradiated to the snout with a single dose of 17 Gy and given a 21-day window to resolve (5 days in addition to the 16-day recovery period determined by measuring the epithelial layer thickness of the tongue following irradiation). On day 21 post-irradiation, animals were then focally re-irradiated to the snout with a single dose of either 12 Gy (the dose necessary to induce radiation-induced OM in ~50% of naive animals) or 17 Gy as before, with euthanasia and tissue harvest conducted on day 11 after the second irradiation. AVA was delivered i.p. as a 24 mg/kg injection 30–60 minutes prior to re-irradiation and then daily until euthanasia. Error bars represent the SEM of the mean, and the statistical unit of analysis was the individual animal.

### Statistical analysis

All statistical analyses were conducted utilizing a two-tailed Student’s t-test with a probability of a type 1 error set to 0.05 as the threshold for significance and a power of 95%. For a detailed breakdown of the number of animals in each group and linked to a specific figure, please refer to **Supplementary Table 1**.

## Results

### AVA increases residual IR-induced DNA damage and micronuclei in NSCLC cells

**Figure 1:**
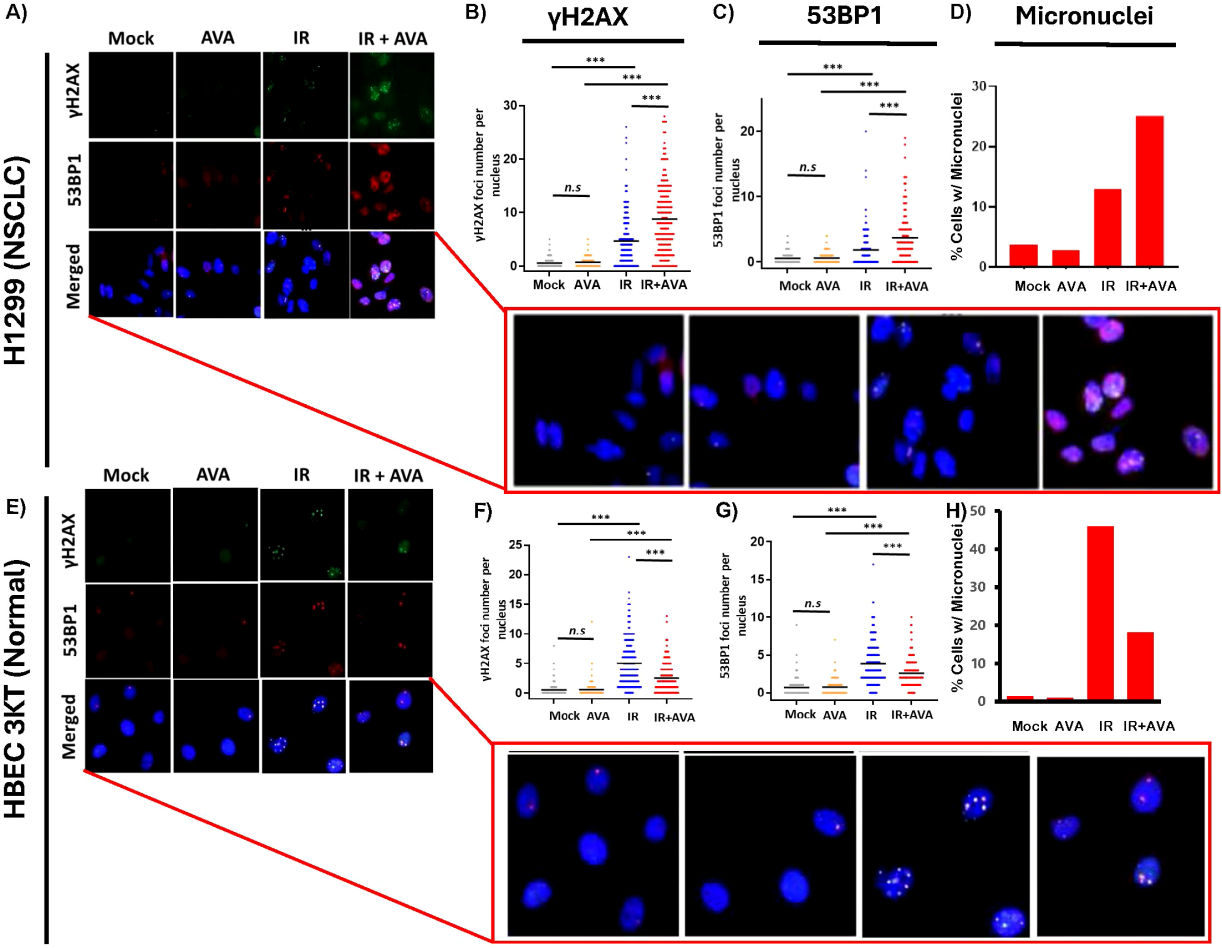
Effect of AVA on DNA repair in H1299 NSCLC cells *vs*. HBEC-3KT immortalized bronchial epithelial cells. **(A)** Representative images of H1299 cells stained with γH2AX (top panel) and 53BP1 (middle panel) and merged images (lower panel) 24 hours post-exposure to 10 Gy γ-rays with and without AVA. **(B)** Quantification of γH2AX foci per cell and **(C)** 53BP1 foci per cell. **(D)** Quantification of the percentage of H1299 cells containing micronuclei. **(E)** Representative images of H1299 cells stained with γH2AX (top panel) and 53BP1 (middle panel) and merged images (lower panel) 24 hours post-exposure to 10 Gy γ-rays with and without AVA. **(F)** Quantification of γH2AX foci per cell and **(G)** 53BP1 foci per cell. **(H)** Quantification of the percentage of HBEC-3KT cells containing micronuclei. AVA, avasopasem manganese; NSCLC, non-small cell lung cancer. ***p < 0.001.

Previously, it was demonstrated that pre-treatment with AVA potentiated high-dose-per-fraction radiation therapy in NSCLC tumors through a mechanism that was dependent upon limited catalase activity in human tumor xenograft models (10). Presumably, this is due to increased DNA damage caused by elevated levels of hydrogen peroxide in the tumor that cannot be detoxified quickly enough by the activity of catalase. To test this hypothesis, H1299 NSCLC cells were irradiated with a single acute dose of 10 Gy with or without a 24-µM dose of AVA present. Staining of γH2AX and 53BP1 DNA damage foci was performed 24 hours post-exposure to detect the presence of residual DNA double-strand breaks. Representative immunofluorescence-stained images are found in **Figure 1A**. γH2AX and 53BP1 foci were significantly increased in the AVA-treated cells determined 24 hours post-IR exposure (**Figures 1B, C**). As further confirmation of an increase in DNA damage, the number of cells with micronuclei in AVA-treated cultures was also higher (**Figure 1D**), confirming an increase in DNA damage, which supports the results from clonogenic survival assays published previously. Representative micronucleus images can be found in **Supplementary Figure 2**.

### AVA reduces residual IR-induced DNA damage and micronuclei in non-tumor HBEC3 KT cells

To determine if the normal tissue radioprotective effect (**Supplementary Figure 1**) is moderated through a reduction in DNA damage (in contrast to the increase observed in H1299 NSCLC cells), immunofluorescence staining to determine the levels of γH2AX and 53BP1 foci was also performed 24 hours post-exposure to IR in cells irradiated with 10 Gy with or without AVA treatment. Representative immunofluorescence-stained images are found in **Figure 1E**. AVA, in contrast to the results observed in the H1299 tumor line, demonstrated significantly reduced numbers of DNA damage foci (**Figures 1F, G**, bottom panel) and numbers of cells with micronuclei (**Figure 1H**, bottom panel), with representative images found in **Supplementary Figure 2**. Therefore, these results indicate a differential response to the combination of IR and AVA treatment in tumor *vs*. normal cells.

### AVA does not increase mutational burden of irradiated cells nor preserve genomic damage

**Figure 2:**
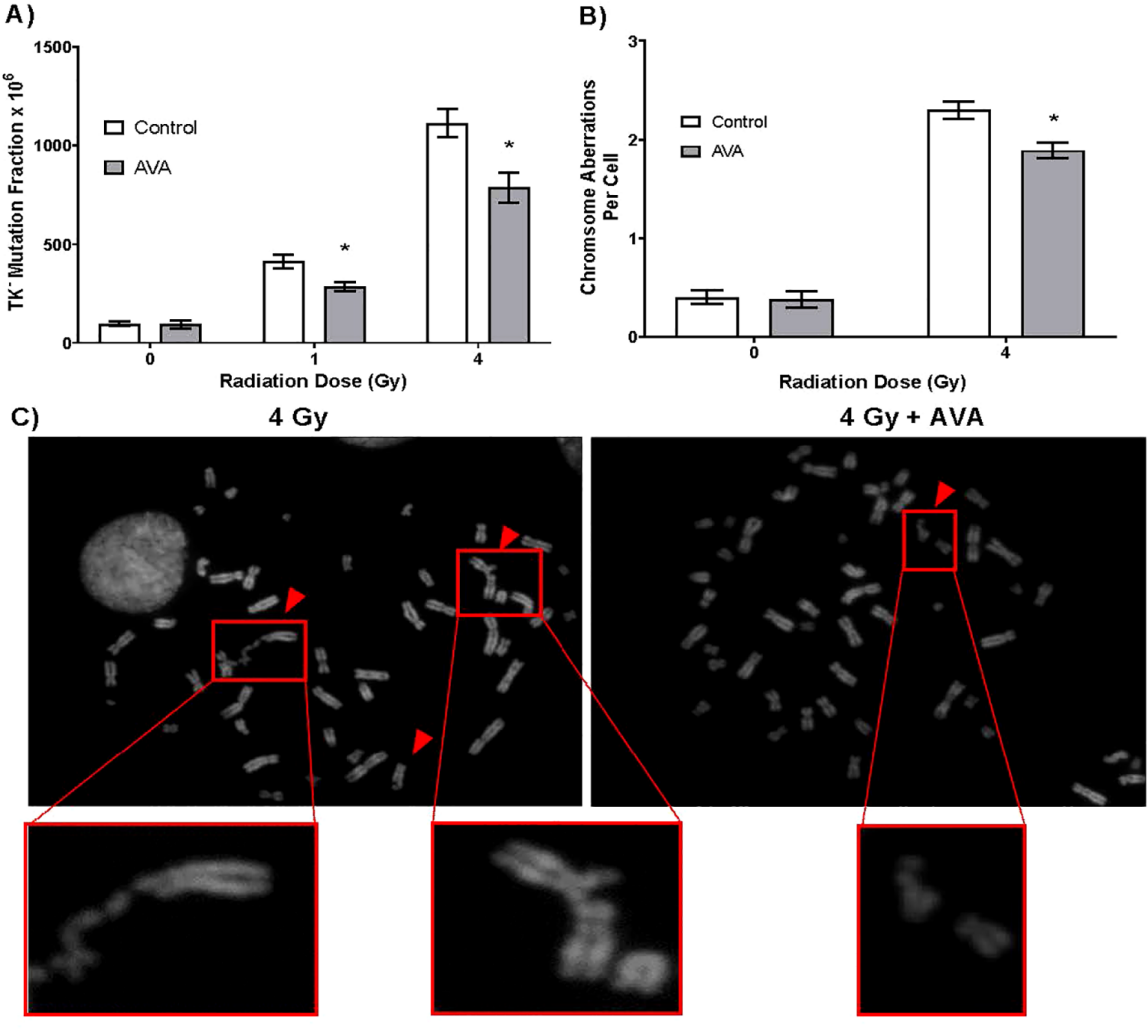
AVA reduces the frequency of mutation and total chromosome aberrations per cell 24 hours post-exposure to IR. **(A)** TK^−/−^ mutant fraction of cells exposed to 1 or 4 Gy γ-rays with or without pretreatment with AVA. **(B)** Frequency of total chromosome abnormalities (both chromosome and chromatid type aberrations) at 24 hours post-exposure to 4 Gy γ-rays. **(C)** Representative images of aberrations in the 4 Gy (left panel) *vs*. 4 Gy + AVA group (right panel). AVA, avasopasem manganese; IR, ionizing radiation. p < 0.05.

To determine the impact of AVA on the radiation-induced mutation frequency in non-oncogenic cells surviving radiation exposure, WTK1 lymphoblastoid cells, commonly used for mutational analysis, were irradiated with doses of either 1 or 4 Gy, which resulted in a dose-dependent increase in TK^−/−^ mutant frequency (**Figure 2A**). However, when AVA was added to the cell cultures 1 hour prior to irradiation and allowed to incubate for 24 hours post-exposure, the observed mutational burden induced by radiation was reduced. Furthermore, cytogenetic analysis of WTK1 cells 24 hours after a single dose of 4 Gy revealed a significantly reduced number of total chromosome (combined chromosome and chromatid) aberrations in irradiated cells treated with AVA (**Figure 2B**). Representative images are also shown (**Figure 2C**).

### AVA reduces severity of radiation-induced lung fibrosis, a late toxicity

The reduced DNA and chromosomal damage, along with the reduced mutational burden in cells treated with AVA, suggested that long-term radiation effects may also be reduced. Therefore, the efficacy of AVA in reducing late effects was tested in preclinical animal models of RILF at doses and treatment schedules approximating the treatment of NSCLC with SAbR.

**Figure 3:**
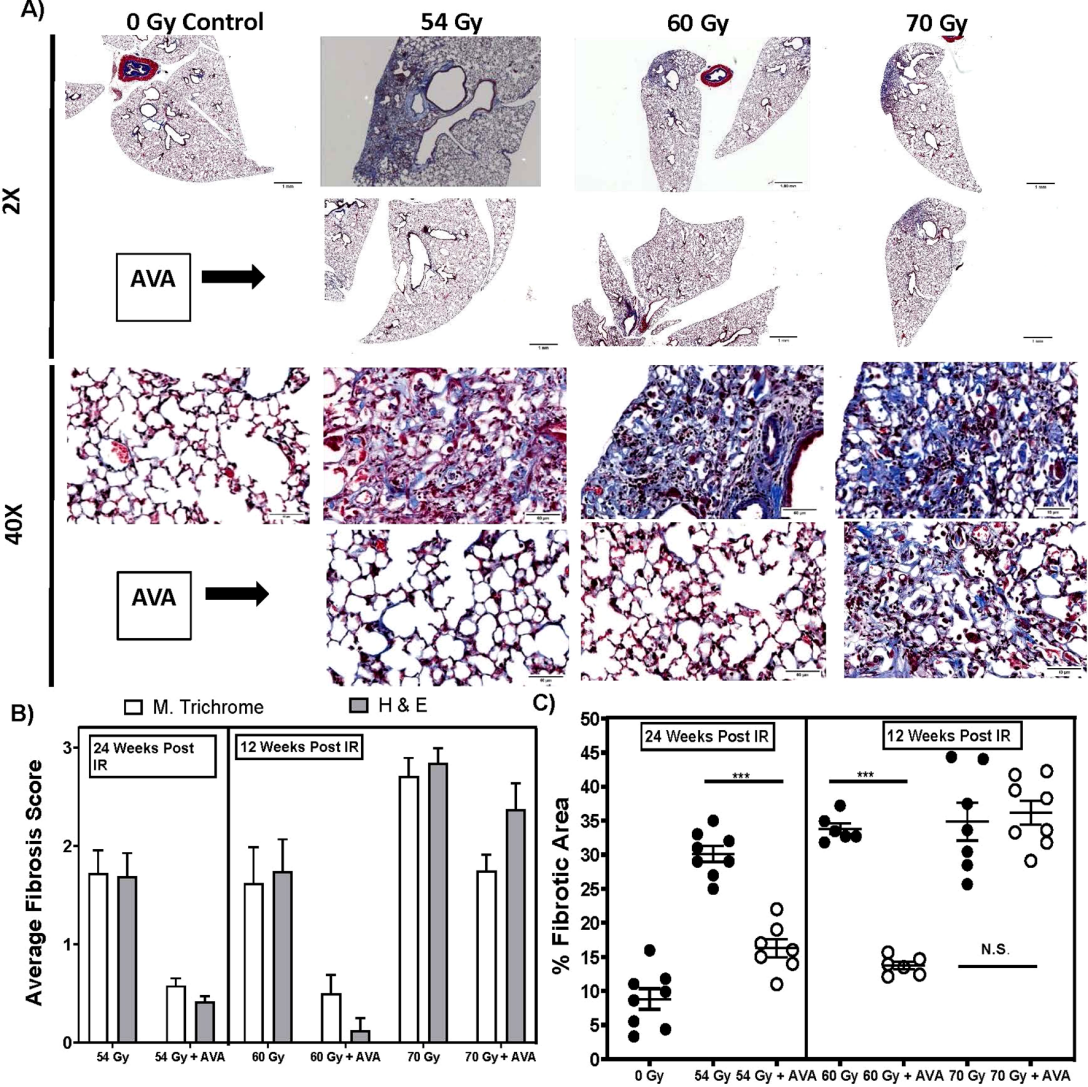
AVA pretreatment reduces the severity of RILF. **(A)** Representative micrographs of MTC-stained lung sections at ×2 and ×40 magnifications in animals exposed to 54 Gy at 24 weeks post-exposure, as well as 60- and 70-Gy sections at 12 weeks post-exposure. **(B)** Quantification of histological sections evaluated by a veterinary pathologist using a modified Ashcroft scale. **(C)** Semi-quantitative image analysis of lung sections measuring the percent fibrotic area across treatment groups. AVA, avasopasem manganese; RILF, radiation-induced lung fibrosis; MTC, Masson’s trichrome. ***p < 0.001.

C57BL/6 mice were irradiated with a single acute dose of 54, 60, or 70 Gy delivered as a vertical 5-mm collimated cone to the left lung, as determined utilizing fluoroscopic imaging. AVA was delivered as a single 24 mg/kg dose 30–60 minutes prior to irradiation. Fibrosis (as detected using Masson’s trichrome staining in histological sections) was examined 24 weeks post-irradiation at 54 Gy and 12 weeks post-exposure in cohorts receiving a higher dose (60, 70, and 80 Gy). Representative images at both ×2 and ×40 magnifications are represented in **Figure 3A**. Pre-treatment with AVA greatly reduced lung fibrosis at 25 weeks in the cohorts treated with 54 Gy and at 12 weeks in the cohorts irradiated with 60 and 70 Gy, as determined using two independent metrics: the pathological score and the modified Ashcroft scale (**Figure 3B**). The fibrotic area, as determined via semi-quantitative image analysis of the irradiated region of the lung, was reduced in the 54- and 60-Gy cohorts (**Figure 3C**). No protective effect of AVA for fibrosis was observed in the 80-Gy cohorts (data not shown). This suggests that fibrosis can be reduced with the application of AVA prior to at least as much as 60 Gy of radiation by either metric, while results for doses above 60 Gy are inconclusive and depend on the assay used to quantify fibrosis.

### AVA functions as an effective radiomitigator of lung fibrosis delivered after irradiation and is more effective with increasing length of treatment

**Figure 4:**
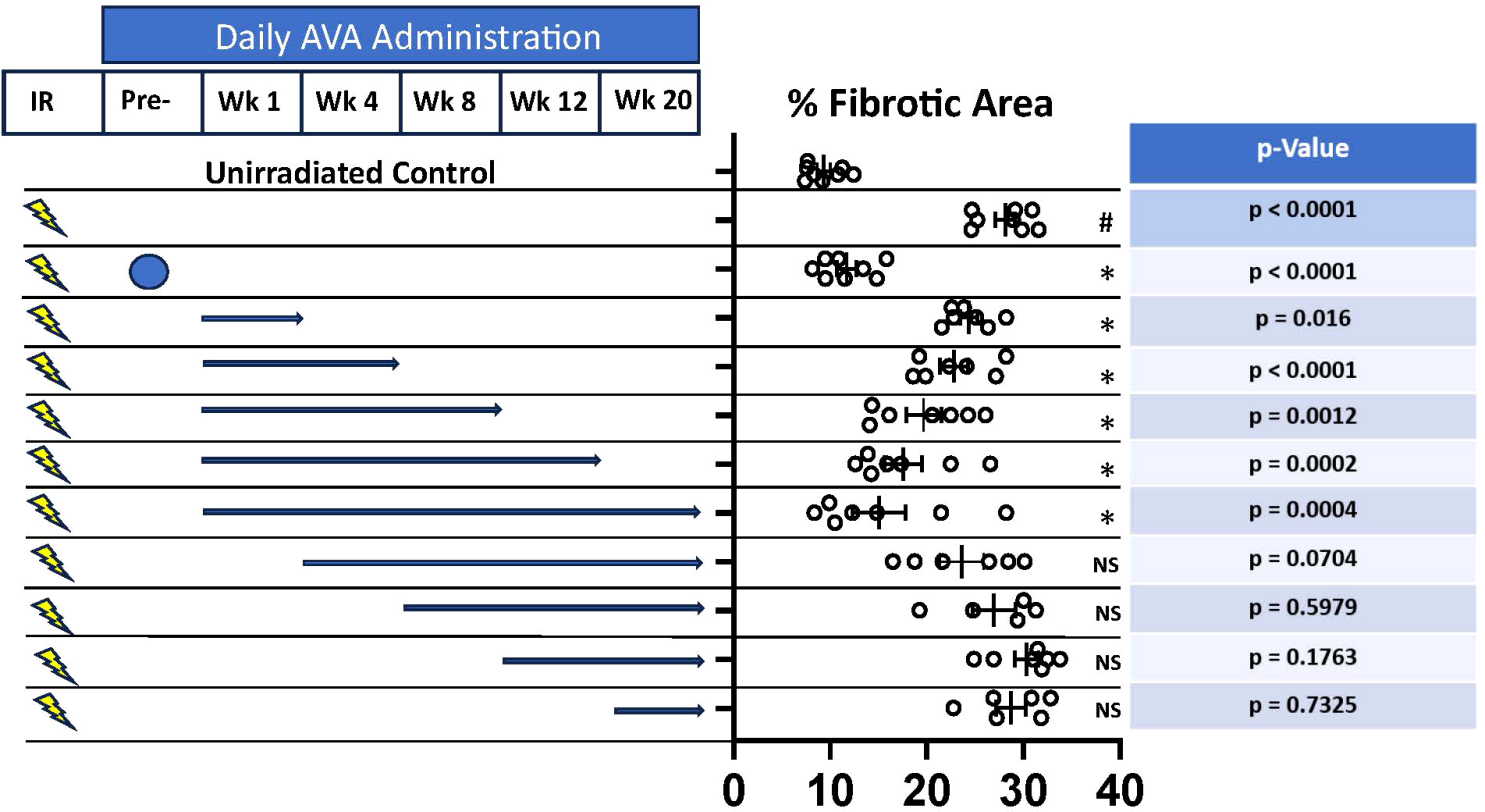
AVA is an effective radiomitigator reducing the severity of RILF. Depiction of AVA and radiation dose delivery schedule (left panel). Blue arrows indicate the length of time daily AVA doses administered at various time points and durations post-exposure. The percent fibrotic area for each group is quantified with significance stated (# = significant when compared against the unirradiated control, * = significant when compared to treatment with 54 Gy alone, and NS = not significant). Finally, p-values are also reported for the appropriate comparisons in the figure (right panel). AVA, avasopasem manganese; RILF, radiation-induced lung fibrosis.

Since AVA reduces superoxide and daughter ROS regardless of source and is effective as a radioprotector in the context of radiation-induced lung fibrosis, whether AVA is also an effective radiomitigator of fibrosis generated by chronically upregulated oxidative stress and inflammatory signaling was tested. Animals were irradiated with a single dose of 54 Gy delivered with a 10-mm collimator to the peripheral lung. AVA dosing schedules started at 24 hours post-irradiation for increasing durations of time or started at later times post-irradiation until 20 weeks post-irradiation. Fibrosis was then determined at 24 weeks post-irradiation for all cohorts. The various dosing schedules of AVA administration are depicted in **Figure 4**, as well as the quantification of the fibrotic area and the relevant p-values to describe statistical significance. These results support the hypothesis that AVA is a radiomitigator when delivered starting 24 hours post-radiation exposure, whose mitigative properties increase as the time of use increases. However, if AVA delivery is delayed, the reduction in fibrosis is reduced and is eliminated if delivery begins 4 weeks or more post-irradiation.

### AVA is both a radioprotector and a radiomitigator of radiation-induced OM of the tongue, an acute (early) effect

**Figure 5:**
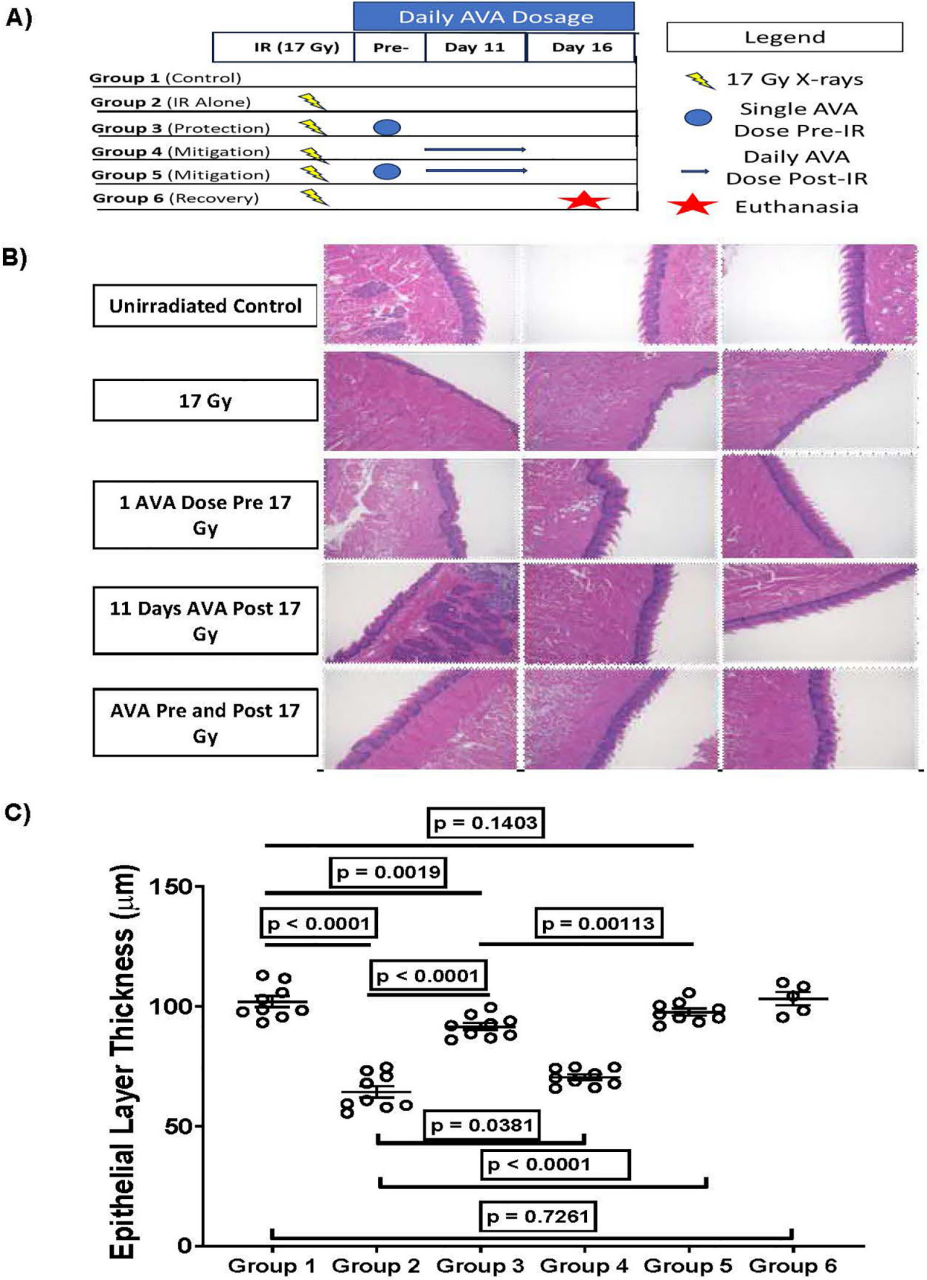
AVA is effective at reducing radiation-induced OM. **(A)** Schematic of treatment schedule used to determine if AVA is effective at reducing OM in mice and group designations. **(B)** Representative micrographs of murine tongues for the various treatment groups exposed to 17-Gy X-rays and varying schedules of AVA administration. **(C)** Quantification of the epithelial layer thickness of tongue mucosa at 11 days post-RT with and without AVA treatment. AVA, avasopasem manganese; OM, oral mucositis; RT, radiotherapy.

AVA demonstrated efficacy as a radioprotector reducing the extent and intensity of radiation-induced OM in clinical trials of patients receiving conventionally fractionated intensity-modulated radiotherapy (IMRT) with cisplatin to treat HNSCC (13, 15) and esophagitis (17). However, because AVA enhanced the response of human HNSCC tumor xenografts to high-dose-per-fraction radiation exposure, whether AVA would be effective in protecting normal oral mucosa of the tongue from ablative radiation exposure (>8 Gy), akin to SAbR regimens sometimes used for HNSCC radiotherapy, was tested in animal models (10). **Figure 5A** describes the schematic of experiments to answer this question, while **Figure 5B** depicts three representative images of the tongue at ×10 magnification from each treatment arm. The quantification of epithelial layer thickness can be observed in **Figure 5C**. Radiation alone significantly reduced the thickness of the epithelial cell layer by an average of ~40% by day 11 post-irradiation (group 1 *vs*. group 2), with the epithelial layer thickness returning to that of the unirradiated epithelial layer by day 16 post-exposure (group 6). AVA was effective as a radioprotector (single dose 30–60 minutes prior to irradiation), significantly ameliorating thinning of the epithelial layer by day 11 post-irradiation (group 2 *vs*. group 3). When AVA was delivered as a radiomitigator (daily AVA dose starting 24 hours post-exposure until euthanasia), it was also effective at ameliorating the reduction in the epithelial layer thickness, albeit much less so than the protection arm (group 2 *vs*. group 4, and group 3 *vs*. group 4). Finally, when AVA was delivered as both a radioprotector and a radiomitigator, a significant reduction in the epithelial layer thickness due to radiation exposure was not observed (group 1 *vs*. group 5). Furthermore, epithelial layer thickness was greater than that of the protection-only arm, indicating that the most effective treatment protocol with AVA for preventing radiation-induced OM in the context of SAbR is as both a protector and a mitigator (group 3 *vs*. group 5).

### AVA is effective at reducing radiation recall of radiation-induced OM

**Figure 6:**
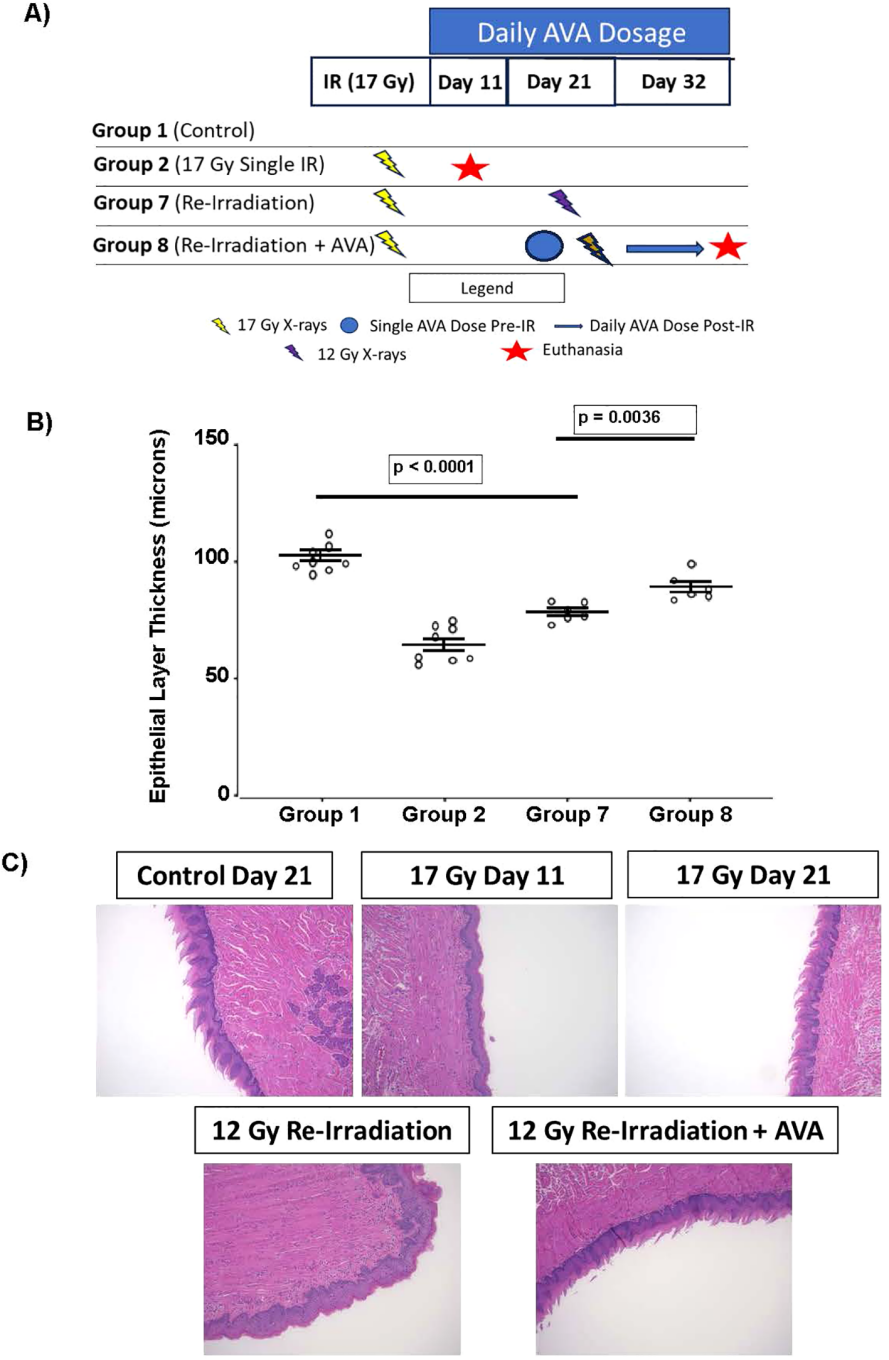
AVA is effective at diminishing the reduction in epithelial layer thickness resulting from IR exposure in the setting of radiation recall. **(A)** Schematic and group designations of animals exposed to generate radiation recall of the oral mucosa. **(B)** Quantification of the thickness of the epithelial layer of groups exposed with or without AVA treatment. **(C)** Representative micrograph images of murine tongue at 11 days after the second dose of radiation. AVA, avasopasem manganese; IR, ionizing radiation.

Re-irradiation can elicit a phenomenon referred to as radiation recall, whereby normal tissues “remember” the original radiation dose and experience severe toxicities. Whether AVA could reduce radiation recall toxicity was also tested in the mouse tongue model with re-irradiation at day 21 after primary irradiation and utilizing the optimal protection + mitigation protocol where AVA was delivered both once prior to the initial irradiation and daily until euthanasia (**Figure 6A**).

Re-irradiation with a second dose of 17 Gy resulted in five of six animals not surviving to 11 days after the second dose, presumably because the severity of OM prevented food intake (data not shown). A re-irradiation dose of 12 Gy, however, was nearly as effective as the single primary dose of 17 Gy in reducing the thickness of the epithelial layer, i.e., recalling the first irradiation, indicating that a second, lower dose of radiation is effective at inducing radiation-induced OM in all animals (group 2 *vs*. group 7). AVA treatment beginning immediately before 12 Gy re-irradiation result significantly attenuated the effects of radiation recall (group 7 *vs*. group 8) (**Figure 6B**). Representative images on ×10 tongue sections can be found in **Figure 6C**.

## Discussion

Radioprotection of normal tissue is not the sole criterion for the use of a compound as a radioprotector or a radiomitigator. Obviously, such a compound could not protect tumors from radiation. An additional requirement to substantiate the long-term efficacy of such a therapeutic is that the compound also has limited or no risk for promoting the survival of cells damaged by radiation and later results in the initiation or promotion of a therapy-induced second tumor. To that end, in *in vitro* experiments, in contrast to effects on cancer cells, AVA reduced the frequency of persistent DNA DSBs and the frequency of micronuclei in normal HBECs while increasing the number of DSBs and micronuclei in tumor cells, presumably indicating additional cell killing without the burden of radiation-induced mutations that have the potential to drive tumor survival or recurrence (**Figure 1**). In further studies investigating mutational burden, particularly in cells that would survive irradiation, AVA reduced the number of radiation-induced TK^−/−^ mutations in lymphoid cells and decreased the frequency of chromosome aberrations, both surrogate markers for carcinogenesis. Taken together, these findings not only confirm prior reports that AVA is an effective radiosensitizer in the context of ablative radiation in NSCLC (10) but also that the radioprotective effects observed in normal tissues also preserve genome integrity, indicating that there is little to no risk of inducing therapy-induced secondary cancers.

Late tissue toxicities, such as RILF, which is of concern for patients undergoing RT to treat lung cancer, breast cancer, HNSCC, or other cancers where fibrosis can be debilitating (e.g., limited lung function, restriction of movements, and cardiotoxicity), are driven by underlying molecular processes such as inflammatory signaling and upregulated oxidative stress driven by metabolic processes. Therefore, effective therapy against RILF would either need to prevent the initial damage from radiation that results in the activation of damaging molecular processes or be capable of mitigating the underlying molecular processes driving the pathology. As the efficacy of AVA in reducing these late effects from RT has not been studied in human cohorts, its efficacy was tested in preclinical animal models of RILF at doses and treatment schedules approximating the treatment of NSCLC with SAbR. The demonstrated functionality in animal models both as a radioprotector and a radiomitigator suggests that whether lung cancer is treated with conventionally fractionated radiation therapy or SAbR, AVA will be effective at reducing the consequences of lung fibrosis (**Figures 3**, **4**). Furthermore, in addition to lung fibrosis, other tissues where the risk for fibrosis may limit the use of fully potent radiation doses may also benefit from AVA. Specifically, these preclinical results imply that optimally, AVA should be delivered prior to RT and then administered as a radiomitigator for an extended period following the completion of treatment. These findings and future clinical trials should seek to elucidate the most effective methodology to employ with regard to radioprotection and mitigation and to modify AVA administration based on the individual patient.

One notable concern is that AVA is delivered intravenously 30–60 minutes prior to irradiation in human clinical trials. Given the extended time course of AVA dosing necessary to observe a reduction in RILF in the preclinical studies presented here, there is a question of the feasibility of delivering AVA for extended periods of time due to the fact that an oral formulation is not currently available. This concern is ameliorated by the fact that in phase III clinical trials, AVA was delivered as a daily intravenous dose prior to each fraction of radiation for up to 7 weeks without observable side effects. Therefore, the long-term delivery of AVA should not be hindered by its method of delivery, pointing to its translational utility as a radio mitigating agent (13, 14, 33).

Not to be minimized is the fact that AVA reduces the mutational burden of cells exposed to radiation, suggesting that AVA may have utility in other radioprotective arenas. The first obvious example is that the radio mitigating properties of AVA may be superior to those of currently used agents, such as Granulocyte-Macrophage Colony-Stimulating Factor (GM-CSF), that stimulate the division of hematopoietic progenitor cells following an environmental exposure to high-dose radiation (such as that experienced in a terrorist attack or a nuclear power plant disaster) to prevent ARS (34). However, it should be noted that this supposition is entirely situation-dependent, as incidents involving accidental radiation exposure have very different doses and exposure scenarios than those commonly encountered in the clinic. Second, given its radioprotective efficacy and good safety profile in clinical trials, there is the potential for orally or subcutaneously available derivatives of AVA to be given as a prophylactic in populations that are exposed to chronic but low-dose-rate occupational radiation such as medical imagining technicians and nurses, uranium miners, airline pilots, radiologists and surgeons using fluoroscopy, laboratory research scientists who work with radioactive isotopes or radiation generating devices, workers at industrial facilities that utilize sealed ionizing radiation sources to sterilize products, or astronauts.

AVA’s recognized efficacy as a clinical radioprotector, reducing the burden of OM in patients undergoing IMRT (2 Gy/fraction at 30–35 daily fractions) concomitant with cisplatin for the treatment of HNSCC, is an example of an acute toxicity where damage to the normal mucosa resulting from treatment leads to ulceration, causing severe pain, malnourishment and dehydration, and even discontinuation of treatment. While there is some movement toward high-dose-per-fraction radiotherapy for HNSCC, SAbR is not a frontline treatment modality for patients with HNSCC but is more commonly utilized as a salvage therapy for patients whose 2-year survival is ~15%. These patients are at risk for radiation recall, which can be devastating (19). The use of a radioprotector/radiomitigator could be highly appropriate and serve as the rationale for examining the protective/mitigating potential of AVA against mucositis with high-dose-per-fraction exposures. Interestingly, while AVA reduced mucositis from single high doses of radiation and mucositis observed with radiation recall (**Figures 5**, 6), AVA is also quite effective at enhancing the response of tumors to radiation therapy when utilized in combination with ablative high-dose-per-fraction radiation exposure akin to SAbR (10). Due to the fact that AVA also protects against radiation-induced OM in the context of high-dose-per-fraction RT, its additional mitigating properties indicate that additional AVA dosage could be given following the completion of RT, in addition to the dosage given prior to irradiation (**Figure 5**).

In summary, AVA, already known to be an effective radioprotector at conventional radiation doses, has now been shown to be an effective protector and now a mitigator of both acute and late effects in the setting of high-dose-per-fraction radiotherapy. Mechanistically, this appears to be in part through a reduction in DNA lesions from mutations to chromosomal and DNA double-strand breaks and a scavenging of ROS generated post-radiation exposure. Combined with its already known anti-tumor properties observed at high-dose-per-fraction radiation exposures, AVA is a highly versatile agent whose application may extend beyond just radiation and into any arena where superoxide is generated for radioprotection or tumoricidal effects.

## Data Availability

The raw data supporting the conclusions of this article will be made available by the authors, without undue reservation.
